# MRI-based shear stress profiles in subclinical carotid atherosclerosis – A feasibility study

**DOI:** 10.1371/journal.pdig.0001618

**Published:** 2026-08-04

**Authors:** Sanne C.M. van Kuijk, Bernhard P. Berghout, Suze-Anne Korteland, Robin Y.R. Camarasa, Marleen de Bruijne, Daniel Bos, Ali C. Akyildiz, M. Kamran Ikram, Jolanda J. Wentzel

**Affiliations:** 1 Department of Epidemiology, Erasmus University Medical Center, Rotterdam, The Netherlands; 2 Department of Cardiology, Biomedical Engineering, Cardiovascular Institute, Thorax Center, Erasmus University Medical Center, Rotterdam, The Netherlands; 3 Department of Radiology and Nuclear Medicine, Erasmus University Medical Center, Rotterdam, The Netherlands; 4 Department of Computer Science, University of Copenhagen, Copenhagen, Denmark; 5 Department of Biomechanical Engineering, Delft University of Technology, Delft, The Netherlands; 6 Department of Neurology, Erasmus University Medical Center, Rotterdam, The Netherlands; Tianjin Medical University General Hospital, CHINA

## Abstract

Arterial wall shear stress (WSS) plays an important role in atherosclerosis, but its assessment is often limited to small, retrospective studies due to technical and time constraints. This study aimed to evaluate the feasibility of WSS assessment in carotid arteries within the long-running, population-based Rotterdam Study. We developed a semi-automated computational fluid dynamics (CFD) pipeline, using deep-learning-based lumen segmentations of the carotid arteries from BlackBlood MRI and participant-specific common carotid blood flow from 3D phase-contrast MRA. Steady-state CFD simulations generated WSS profiles of the common and internal carotid arteries. Feasibility was evaluated by applying the pipeline to both carotid arteries in a sample of the Rotterdam Study, and reliability was assessed through inter- and intra-rater analyses. Simulations were successfully performed on both carotid arteries of 100 participants (mean age, 72.6 years ± 10.6 [SD]; 49 female). Automated lumen segmentations showed good agreement with manual segmentations (Dice similarity coefficient 0.89 ± 0.02). Intra- and inter-rater reliability analyses indicated good to excellent reliability of the pipeline, with intra-class correlation coefficients ranging from 0.89 to 0.98. This study demonstrates the feasibility of the CFD-based pipeline for large-scale carotid WSS assessment, integrating participant-specific carotid blood flow and automated segmentations. The reliability analyses underscore the reproducibility of the pipeline and highlight its potential for efficient, scalable carotid WSS analysis, making the pipeline suitable for application in large population-based and clinical studies.

## Author summary

Arterial wall shear stress (WSS) is the tangential component of the force exerted on the arterial wall due to blood pressure. It is believed that WSS plays an important role in the development of atherosclerosis, a major contributor to stroke and cardiovascular disease. However, studying WSS in large populations has been challenging due to the time-consuming and technical nature of such studies. In this study, we addressed this challenge by developing a semi-automated computational fluid dynamics based pipeline that assesses WSS in the carotid arteries using magnetic resonance imaging. Our approach combines automated carotid segmentations and carotid specific blood flow measurements to perform individualized simulations. We demonstrated that the pipeline can be applied efficiently and reliably, producing consistent results while minimizing workload. Furthermore, we demonstrated the applicability of the pipeline by applying it on both carotid arteries of 100 participants. This pipeline enables assessment of carotid WSS on a population level, which may provide valuable insights into the role of carotid hemodynamics in cardiovascular pathologies in future studies.

## Introduction

Hemodynamic forces play an important role in vascular pathology. As blood flows through the arteries, a pressure is generated that exerts a perpendicular force on the arterial wall. In addition, the tangential component of this force, the arterial wall shear stress (WSS), is sensed by the endothelium through a shearing deformation. WSS plays a crucial role in regulating endothelial functioning and vascular remodeling, and it is associated with the development of atherosclerosis. Throughout the pathophysiology of atherosclerosis, different magnitudes of WSS drive distinct endothelial responses, underscoring the complex interplay between WSS and endothelial functioning [1]. However, despite its importance, our understanding of in-vivo WSS remains limited as most human studies have either been underpowered or relied on retrospective analyses of imaging acquired in specific clinical trials [1–3]. Consequently, population-based estimations of WSS are missing, leaving a gap in our understanding of its variability across individuals and its role in atherosclerosis development.

Local WSS may be directly derived from 4D flow MRI. However, 4D flow MRI is rarely available in large population-based studies with long follow-up times, a limitation that is common across medical imaging techniques (e.g., CT, X-ray, PET scans) [4]. Additionally, the relatively low spatial resolution of 4D flow MRI can compromise the accuracy of WSS measurements [5]. Instead, WSS is often computed from 3D anatomical reconstructions of MRI or CTA based imaging, through the use of simulations based on computational fluid dynamics (CFD) [1,6]. These simulations are often carried out with boundary conditions retrieved from literature averages, rather than participant-specific measurements, which limit their validity [7]. Moreover, the computational demands and time-consuming nature of CFD studies make it impractical for large-scale population studies.

To address these limitations, we developed a semi-automated CFD-based pipeline designed to derive participant specific WSS profiles of the carotid arteries, tailored for application in a large population-based, prospective cohort study. This study is an important step toward bridging the gap between computational engineering and large-scale population-based epidemiological studies. This approach will allow us to study WSS at a population-level in the near future, enabling us to draw meaningful conclusions about the role of WSS in vascular health. In the current study, the reliability of the CFD-based pipeline is evaluated to support its use in future large-scale analyses. Furthermore, we present an initial application of this pipeline by comparing WSS metrics of the left and right carotid arteries.

## Materials and methods

### Study setting

This study is embedded within the Rotterdam Study, an on-going, population-based observational cohort study of residents aged ≥45 years from the Ommoord district of Rotterdam, the Netherlands. Further details of this study are described elsewhere [8]. Briefly, between 2007 and 2012, 2666 participants were identified with an intima-media thickness ≥2.5 mm in one or both carotid arteries through ultrasonography examination. These participants were subsequently invited to undergo MRI examination of their carotid arteries.

Of the invited participants, 684 were not scanned due to the following reasons: 272 declined MRI examination, 191 were unable to undergo scanning due to physical constraints, 115 had contraindications to MRI, 57 participants were claustrophobic, and 49 either did not show up or were lost to follow-up. Among the 1982 participants who were scanned, 106 scans were interrupted and 95 were of insufficient quality, leaving a study population of 1781 participants [9].

### Ethics statement

The Rotterdam Study has been approved by the Medical Ethics Committee of the Erasmus MC (registration number MEC 02.1015) and by the Dutch Ministry of Health, Welfare and Sport (Population Screening Act WBO, license number 1071272–159521-PG). All participants provided written informed consent to participate in the study and to have their information obtained from treating physicians.

### Imaging settings and acquisition

Imaging of carotid arteries was performed using a 1.5-Tesla MR scanner (GE Healthcare, Milwaukee, USA) with a bilateral phased-array surface coil (Machnet, Eelde, the Netherlands) [10]. Participants were stabilized in a custom-designed head pouch to reduce motion artefacts. High-resolution MR images were obtained through a previously described protocol [10], consisting of the following steps. First, both carotid bifurcations were identified with 2-dimensional time-of-flight MR angiography, ranging from 15mm caudally to 30mm cranially from the bifurcation [11]. After this, several high-resolution MRI sequences were obtained, of which the following were used: a proton density-weighted fast spin echo black-blood (PDw-FSE-BB) sequence with increased in-plane resolution and a 3D phase-contrast MR angiography (PC-MRA).

Of the 1781 participants, 330 did not have either the PDw-FSE-BB or the PC-MRA sequence available. As a result, MRI scans of both carotids were available for further research on carotid hemodynamics for 1451 participants.

### Image processing

The following steps outline how carotid MR images were converted into a 3D reconstruction of the carotid arteries and a finite element mesh, suitable for CFD simulations of local carotid WSS profiles.

First, axial PDw-FSE-BB images were used as a base input for a nnU-Net [12] deep-learning segmentation method [13] to automatically construct lumen segmentations of the common (CCA) and internal carotid arteries (ICA). Next, the resulting segmentations were prepared for CFD analysis using the software programs ‘3D Slicer’ [14] and ‘ANSYS SpaceClaim’. Cylindrical flow extensions were added to the carotid artery inlets to ensure fully developed flow and a circular inlet profile. Further details on the preparation of the segmentations for CFD analysis are outlined in the S1 Text. Finally, the geometries were converted into 3D tetrahedral finite-element meshes suitable for CFD simulations using ANSYS ICEM software (version 2022.R2). To accurately calculate steep velocity gradients near the vessel wall, a five-fold prism layer was applied at the lumen-wall boundaries. The number of elements ranged between 6.4*10^5^ and 2.7*10^6^, with an average element size of 1.1*10^-3^ mm^2^. Additional details about determining the most optimal mesh size are available in the S2 Text.

### Boundary conditions

For the CFD simulations it is required to have blood flow as an input boundary condition. Therefore, common carotid blood flows were measured on 3D PC-MRA images, 22.5 mm proximal from the bifurcation, as described in previous work [15,16]. A luminal centerline was first generated, along which luminal cross-sectional areas were manually segmented. The inlet flow was determined as the average flow across three consecutive cross-sections. An in-depth description of this methodology is available in the S3 Text. The measured inflow was applied using a parabolic velocity profile at the extended inlet of the common carotid 3D mesh. As outflow boundary conditions at the outlets of the internal and external carotid arteries (ECA), a ratio was used dependent on the degree of stenosis in the ICA as described previously [15]. For cases with area stenosis ≤64%, the ICA:ECA flow ratio remained close to 0.64:0.36, with a gradual linear decline as stenosis severity increased. After 64% stenosis, the decrease in ICA flow became more pronounced [15].

### CFD simulations

Steady-state CFD simulations of blood flow through the carotid arteries were performed using ANSYS Fluent (version 2022.R2). In this software, blood was modeled as a non-Newtonian incompressible fluid, using a Carreau viscosity model (settings: density 1060 kg/m^3^, time constant 25 seconds, power law index 0.25, zero shear viscosity 0.25 kg/ms, infinite shear viscosity 3.5*10^-3^ kg/ms). The walls were modeled as rigid with a no-slip boundary condition [17].

### Post-processing and analysis of simulation output

**Fig 1:**
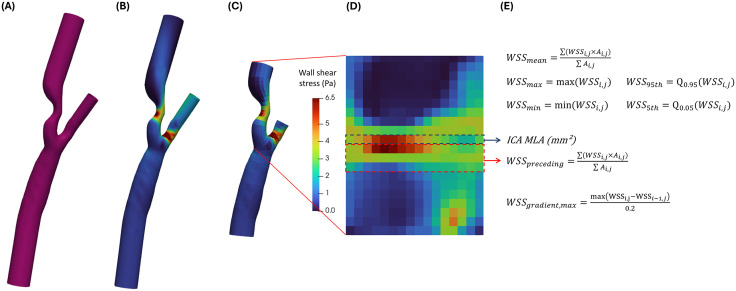
Illustrative example of turning a 3D carotid geometry into a 2D heatmap of wall shear stress averaged into circumferential bins for further analysis. (A) depicts the segmentation after completing the 3D Slicer steps; (B) depicts the result of the CFD simulation; (C) depicts the binned shear stress maps of the carotid artery after removal of the artificial cylindrical extensions; (D) depicts the 2D WSS heatmap of the ICA; and (E) shows how the different WSS metrics have been determined with *WSS*_*i,j*_ and *A*_*i,j*_ referring to the WSS value and area of one patch respectively.

The CFD simulations produced 3D maps of the WSS across the bifurcations, illustrated in Fig 1. WSS data from these simulations was averaged over 16 circumferential bins of 1.0 mm in length (22.5°x1.0 mm) for the CCAs and ICAs separately, and plotted on 2D heatmaps. At the minimum lumen area (MLA) within the ICA, the data was obtained in a higher resolution using 16 circumferential bins of 0.2 mm in length (22.5°x0.2 mm). This high-resolution profile enabled measuring the longitudinal spatial WSS gradient, by subtracting the WSS of the subsequent bin from the prior one and dividing by 0.2 mm.

From the 2D heatmaps with bins of 1.0 mm in length, mean, minimum, 5^th^ percentile, maximum, and 95^th^ percentile WSS were assessed per artery. Furthermore, the mean WSS in the first 3 mm proximal to the MLA was assessed.

### Sample selection and performance

The CFD-based pipeline was applied to a consecutive sample of participants from the Rotterdam Study cohort to evaluate its performance. Participants with prevalent stroke at the moment of MR imaging were excluded from the sample. The pipeline was performed until CFD simulations were successfully completed for both the left and right carotid arteries for 100 participants, ensuring bilateral data availability. In another 20 randomly selected carotid arteries, the median runtime for the finalized pipeline was recorded on a 13^th^ Gen Intel® Core™ I7-13700 system, using 14 cores and 32GB of RAM.

### Statistical analyses

Baseline characteristics of the study sample were summarized using means and standard deviations for continuous variables, and frequencies and percentages for categorical variables.

Intra- and inter-rater reliability assessments were conducted to evaluate the consistency of the common carotid blood flow measurements and the WSS output of the complete CFD-based pipeline. Outcome measures to assess this were the common carotid inlet flow and the mean WSS in both the CCA and ICA, as these are key outcomes of the pipeline and might be influenced by manual corrections made while performing the pipeline. The assessments were performed on a randomly selected subsample of 30 carotid arteries. The inter-rater reliability was performed by calculating the intra-class correlation coefficient (ICC) among measurements done by two individual raters (author SvK and a research assistant). Additionally, each rater repeated their measurements to assess the intra-rater reliability. For all reliability assessments, the two-way mixed effects ICC was calculated.

To validate the accuracy of the automated carotid segmentations, Dice similarity coefficients were calculated by comparing them with manually created carotid segmentations using the Segment Comparison Module in 3D Slicer. This analysis was also performed on a randomly selected subgroup of participants.

The complete study sample of 100 participants was used to demonstrate the applicability of the pipeline for analyzing carotid biomechanics. Differences in parameters of artery geometry, flow, and WSS between left and right carotid arteries were assessed using two-sided tests with t-tests for normally distributed continuous variables or Wilcoxon signed-rank tests for non-normally distributed continuous variables. To evaluate whether observed differences in WSS were explained by geometric or flow-related asymmetries, generalized linear mixed-effects models were fitted using a Gamma distribution with a log link. In Model 1, the WSS metrics were included as the dependent variables, with carotid side and average diameter of the corresponding carotid segment as fixed effects. Model 2 additionally adjusted for the flow of the respective carotid segment. Pairwise comparisons were performed to assess side differences and are reported as ratios (left/right).

All statistical analyses were performed in R, version 4.4.1, using the packages “psych” (v2.4.12) and “ggpubr” (v0.6.0).

## Results

Characteristics from the selected 100 participants are presented in Table 1. The median age at the time of imaging was 76 years (IQR: 62–80 years), and 49% were female.

**Table 1:** Participant characteristics of the sample (n = 100).

Characteristic	Mean/Count (SD/%)	Missing %
Age at MRI imaging, years	72.6 (10.6)	0
Sex, female	49 (49)	0
Hypertension	69 (69)	13
Smoking, current	15 (15)	12
Dyslipidemia	64 (64)	15

To obtain bilateral data from 100 participants, the CFD-based pipeline was performed on 306 carotid arteries from 153 participants. While running the pipeline, 17% of the carotid arteries were excluded due to insufficient MR imaging quality or an insufficient region of interest. Furthermore, 7% of carotids were not available due to inaccurate flow calculations or errors during meshing and post-processing. For this study, only those participants for whom bilateral data was available were included. Among the 53 participants for whom this was not available, the left carotid was lost in 10 participants, the right carotid in 24 participants, and for 19 participants the simulation could not be completed for both carotid arteries.

Corrections to the automated segmentation, including adjustments to the segmentation contour and extension of the segmentation when full MRI coverage was not initially included, were required in 12 of 200 carotid arteries (6%).

The median time required for the pipeline to measure flows was 61 (range: 55–82) seconds. Furthermore, preparing the carotid segmentation for CFD analysis was complete in a median time of 136 (range: 106–190) seconds. The median computational runtime, including meshing and running Ansys Fluent, was 415 (range: 343–745) seconds per carotid artery.

### Intra- and inter-rater reliability

Table 2 presents the intra- and inter-rater reliability assessments of the common carotid blood flow measurements and the entire CFD-based pipeline, all evaluated in 30 carotid arteries. The reliability assessments resulted in ICC values ranging from 0.89 to 0.98, indicating good to excellent reliability across and within raters.

**Table 2:** Intra- and inter-rater reliability assessments (ICC [95% CI]).

	Common carotid blood flow	CCA mean WSS	ICA mean WSS
Inter-rater ICC	0.89 [0.79-0.95]	0.92 [0.84-0.96]	0.89 [0.78-0.95]
Intra-rater ICC, rater 1	0.92 [0.84-0.96]	0.97 [0.94-0.99]	0.98 [0.95-0.99]
Intra-rater ICC, rater 2	0.95 [0.91-0.98]	0.96 [0.91-0.98]	0.94 [0.88-0.97]

Abbreviations: CCA = common carotid artery, ICA = internal carotid artery, WSS = wall shear stress, ICC = intra-class correlation coefficient.

### Automated segmentation agreement

The Dice similarity coefficients were calculated for 10 carotid geometries for which the segmentations were created both manually and through the automated method. An average Dice similarity coefficient of 0.89 ± 0.02 was found, suggesting good agreement between the manual and automated segmentations.

### WSS

S1 Table depicts the differences in WSS metrics between the left and right carotid arteries. In general, shear stress values were higher in the left carotid arteries compared to the right. Significant differences were observed between the high-end WSS metrics (95^th^ percentile and maximum) in both carotid segments, as well as the mean WSS in the CCA, with differences ranging from 15-36% across carotid regions and WSS metrics.

No differences between CCA and ICA inlet flow were observed. Furthermore, the average CCA diameter was found to be larger for the right carotid arteries, whereas the average ICA diameter was larger for the left carotid arteries.

After adjustment for carotid diameter using mixed-effects models, differences in WSS between the left and right carotid arteries persisted, although their magnitude was reduced (S2 Table). Furthermore, significant differences were observed between all other WSS metrics as well, with the exception of minimum WSS in the ICA. When additionally adjusting for flow, the observed differences in the ICA remained, whereas significant differences in the CCA only persisted for the low-end WSS metrics (minimum and 5^th^ percentile).

## Discussion

In this study, we established a semi-automated pipeline to measure WSS in carotid arteries and assessed that both inter- and intra-rater reliability showed good to excellent agreement, underscoring the robustness of the pipeline. By integrating all components of the CFD process into a single pipeline, the workflow is streamlined, making carotid WSS assessment more time-efficient and accessible for large-scale use in population and large patient cohort studies. Preliminary analysis of WSS measurements, performed in a sample of 100 participants, demonstrated the feasibility of acquiring WSS on a large scale.

Previous studies investigating carotid hemodynamics have shown heterogeneity in terms of sample size, study design, imaging modalities, and computational approaches. Most studies include small cohorts that only incorporate up to a few dozens of participants, with limited studies exceeding 100 individuals. Moreover, differences in study design and computational setup have restricted the generalizability of the findings, underscoring the need for larger prospective studies to validate previously determined relationships between WSS and vascular pathologies [18].

The WSS assessment within our pipeline using CFD, relies on both the derived local lumen geometry and the measured flow. The presented CFD pipeline combined previous work on MRI-based lumen, wall, and plaque composition assessment, which have been extensively used and validated in prior publications [9,15,16]. Furthermore, it uses automated segmentations of carotid artery lumens through the use of the deep-learning nnU-Net technique [12]. The accuracy of the automated segmentations was validated by comparison to manually created segmentations in this study. The Dice similarity score demonstrated excellent agreement, supporting the reliability of the automated method. Collectively, these components underscore the validity of the developed pipeline to assess WSS on a population level.

The CFD results from the 100 participants suggest a potential difference in WSS parameters between the left and right carotid arteries. To the best of our knowledge, such differences in WSS have not been previously reported. A few studies have explored differences between left and right carotid arteries in terms of geometry, flow and plaque composition, finding larger IMT, higher blood flow and more intraplaque hemorrhage in the left carotid arteries compared to the right [19–21]. These findings may indicate underlying asymmetries in carotid hemodynamics, which could be related to the observed differences in WSS. Within our 100 participants, no flow difference between the left and right carotid arteries were observed. However, geometrical asymmetries were identified. The right CCA exhibited larger diameters compared to the left, whereas the left ICA was larger compared to the right. Because WSS is inversely related to vessel diameter, the larger right CCA may partially explain the lower mean and maximum WSS observed on that side. However, after adjusting for diameter in mixed-effects models the differences in WSS between sides remained, although attenuated. By additionally adjusting for flow, the observed differences remained in the ICA. This indicates that these side differences cannot be explained by diameter and flow alone. Further investigation in a larger cohort is required to explore these differences more in depth.

A follow-up study is planned within the full Rotterdam Study cohort, to extensively investigate WSS in all participants, stratified by sex, age groups, and plaque phenotype based on carotid MR imaging. This will help establish normal ranges of WSS and identify determinants influencing carotid WSS in males and females across different ages. Furthermore, it will enable the exploration of associations between WSS and neurovascular pathologies such as stroke, TIA, and dementia.

### Strengths and limitations

The strength of this study lies in the robustness of the pipeline. Reliability assessments showed good to excellent agreement across both intra- and inter-rater evaluations, indicating the consistency of the blood flow calculations and complete pipeline outputs. Furthermore, applying the carotid specific automated segmentations and blood flow measurements enhances the validity of the results, as this eliminates the often used literature averages for boundary conditions and idealized geometries. Additionally, it has been shown that the pipeline can be applied on a large scale and to carotid arteries of individuals across a broad age range, with varying degrees of stenosis and calcification. This enables the quantification of WSS profiles of carotid arteries within population-based and large patient cohort studies. This methodology thereby offers a distinct advantage in obtaining participant-specific WSS parameters over the current existing literature from predominantly retrospective, small-number patient studies. Although the pipeline was evaluated in a population with subclinical atherosclerosis and varying degrees of disease severity, further validation in populations with advanced atherosclerotic lesions is warranted to confirm the applicability of the pipeline in these settings.

Besides these strengths, several limitations should be acknowledged. To successfully collect CFD results for both carotid arteries in 100 participants, the pipeline had to be applied to 153 participants. Most of the unsuccessful cases were due to insufficient imaging quality or an incorrect region of interest of the carotid, factors independent of the CFD-based pipeline. Although a standardized imaging protocol was used, occasional manual adjustments to coil positioning were required to effectively image the carotid arteries. In some cases, this resulted in exclusion of the carotid bifurcation or an insufficient length of the CCA for reliable flow determination or geometry reconstruction, leading to an insufficient region of interest. Furthermore, as this study aimed to compare left and right carotid arteries, successful completion for both sides was required to include a participant. If such a comparison was not performed, only 19 of the 153 participants would have been excluded completely, demonstrating that the pipeline can be applied successfully in large cohorts.

Moreover, although this study relies on several extensively validated methods, a potential source of measurement error is the measurements of the mean common carotid artery blood flow, derived from 3D PC-MRA images. The measured blood flows represent the mean flow over the duration of the MRI scan, which complicates comparisons with the literature, where blood flow is mostly reported as peak-systolic or end-diastolic velocity. Nevertheless, when compared with the scarce literature available, our estimates of the mean common carotid blood flow are comparable with previous reports that range between 2.5-7.4 ml/s [22,23], suggesting that our measurements are likely accurate.

Another limitation of this study is the use of steady-state CFD simulations rather than time-resolved simulations. As a result, dynamic WSS parameters, such as the oscillatory shear index or relative residence time, could not be assessed. On the other hand, steady-state WSS estimates have been shown to provide reliable approximations of time-averaged WSS [24], making them suitable for studies where the focus is on hemodynamic loading, rather than on temporal flow characteristics. Furthermore, hemodynamic parameters derived from steady-state simulations have been linked to pathologies like atherosclerosis, making them valuable parameters to study [6]. Moreover, focusing on steady-state simulations significantly reduces computational demands, making it feasible to perform carotid WSS analyses in a large, population-based study.

In the current study, steady-state simulations were performed due to the available flow measurements. These represented the average flow over the duration of the MRI scan and therefore did not provide the time-resolved boundary conditions required for transient CFD analyses. Future studies incorporating time-resolved flow measurements could enable transient simulations. Moreover, AI-based approaches might offer opportunities to extend steady-state simulations towards time-resolved ones [25], for which the current pipeline could serve as a foundation.

Finally, the minimal manual steps required to prepare the segmentations for CFD analysis could introduce some residual variance in WSS measurements. However, most of these manual adjustments are excluded from the simulation output and are thus unlikely to critically impact the results. This was further confirmed by intra- and inter-rater reliability assessments of the complete pipeline, supporting the conclusion that these manual adjustments have a minor effect on the findings.

To conclude, we developed a pipeline to assess carotid WSS, specifically designed for large population-based and clinical studies using MR imaging. By including automated deep-learning segmentation methods and participant-specific flow measurements, this pipeline enhances the accuracy, efficiency and scalability of carotid WSS analysis. Currently, this pipeline is being applied in the long-running Rotterdam Study, in order to explore the variability of carotid WSS among individuals and assess its effects on neurovascular diseases.

## Supporting information

S1 Text Description of carotid geometry preparation. (DOCX)

S2 Text Description of mesh refinement study. (DOCX)

S3 Text Description of blood flow measurements. (DOCX)

S1 Table Comparison of measurements between 100 left and 100 right carotid arteries. (DOCX)

S2 Table Pairwise comparison of WSS metrics between 100 left and 100 right carotid arteries, adjusted for carotid diameter and flow. (DOCX)
