# Supplementary material for: MRI-based shear stress profiles in subclinical carotid atherosclerosis – A feasibility study
Source: PLOS Digit Health. 2026 Aug 4;5(8):e0001618. doi: 10.1371/journal.pdig.0001618 (PMC13436755; doi:10.1371/journal.pdig.0001618)
Supplement: Supplementary file 1 [file pdig.0001618.s001.docx]

**S1 File. Description of carotid geometry preparation**

Previous work within the Rotterdam Study employed the ‘nnU-Net’ (version 2.0) software, a well-established and fully automated, deep learning-based segmentation method, to obtain segmentations of the lumen and walls of the common and internal carotid arteries. From the 1781 participants who underwent MR imaging of their carotid arteries, scans from 82 participants were randomly selected. This subset was divided into a training set (40%), a validation set (10%), and an independent test set (50%). The nnU-Net software was then trained and validated using this subset. After the results from the test set were visually deemed satisfactory, the remaining 1740 participants were successfully segmented.

The 3D Slicer (version 5.2.1) software was used to complete the automated lumen segmentations through the following steps: 1) manual correction of segmentation errors; 2) extending the segmentation approximately 5 mm into the external carotid artery through manual delineation of the lumen starting from the carotid bifurcation; 3) initial smoothing of the voxel-based segmentations into a realistic smooth representation of the lumen, using a smoothing factor of 0.3 within 3D slicer. Lastly, 4) opening the segmentations by cutting the in- and outlets normal to the centerline, and adding cylindrical flow extensions to all ends. These extensions, each with a length equal to three to five times the vessel radius, ensure fully developed flow at the model boundaries. This geometry then underwent a final smoothing procedure in ANSYS SpaceClaim (version 2022.R2), where additional facets were first applied to the whole geometry with an angle threshold of 35°, and ‘volume aware’ smoothing was applied with an angle threshold of 70°.
