# Supplementary material for: MRI-based shear stress profiles in subclinical carotid atherosclerosis – A feasibility study
Source: PLOS Digit Health. 2026 Aug 4;5(8):e0001618. doi: 10.1371/journal.pdig.0001618 (PMC13436755; doi:10.1371/journal.pdig.0001618)
Supplement: Supplementary file 2 [file pdig.0001618.s002.docx]

**S2 File. Description of mesh refinement study**

To determine the optimal element size, a mesh refinement study was performed on six carotid arteries, balancing computational efficiency and the integrity of results derived from subsequent steady-state CFD simulations. To that end, eight different scale factors were applied to adjust the overall size of each mesh. From coarse to fine, these scale factors were 8.0, 4.0, 2.0, 1.0, 0.5, 0.3, 0.2, and 0.15.

Per carotid, the number of elements was plotted against the area-weighted WSS, which was derived by the following formula:

$$\bar{WSS}=\frac{\sum_{i=1}^{n} (WSS_{i}*A_{i})}{\sum_{i=1}^{n} A_{i}}$$

Figure S1 illustrates the mesh refinement results of one carotid artery, representative for all carotids. Based on these plots, it was determined that a scale factor of 0.3 is the most suitable for the mesh conversion. This corresponds with an average element size of 1.1*10^-3^ mm^2^.

**Figure S1. Variation of area-weighted WSS (Pa) and the corresponding offset (%) with mesh refinement. The offset represents the percentage deviation from the WSS value obtained at a scale factor of 0.15. Scale factors are annotated along the WSS curve.**
