# Supplementary material for: MRI-based shear stress profiles in subclinical carotid atherosclerosis – A feasibility study
Source: PLOS Digit Health. 2026 Aug 4;5(8):e0001618. doi: 10.1371/journal.pdig.0001618 (PMC13436755; doi:10.1371/journal.pdig.0001618)
Supplement: Supplementary file 3 [file pdig.0001618.s003.docx]

**S3 File. Description of blood flow measurements**

The non-triggered 3D PC-MRA sequences were used to measure blood flow in common carotid arteries (Q_CCA_), where a mean Q_CCA_ was determined over the entire scan duration for each artery separately. To that end, the following settings were applied to the scanning system: repetition time/time to echo = 12,000 ms/24.3 ms, field of view = 18x18 cm^2^, in-plane resolution = 0.70 mm, slice thickness = 1.0 mm, scan time = 373 seconds, and velocity scale factor *k* = 3.44, with a three-directional velocity encoding (VENC) of 60x60x60 cm/s. The following formulas provided by the scanner manufacturer were used to compute local velocities in the x, y, and z directions:

$$v_{x}=\left( \frac{S_{px,lumen}-S_{px,stat}}{S_{mag,lumen}} \right)\frac{v_{enc}}{\pi k}$$

$$v_{y}=\left( \frac{S_{py,lumen}-S_{py,stat}}{S_{mag,lumen}} \right)\frac{v_{enc}}{\pi k}$$

$$v_{z}=\left( \frac{S_{pz,lumen}-S_{pz,stat}}{S_{mag,lumen}} \right)\frac{v_{enc}}{\pi k}$$

In this context, S_px,lumen_, S_py,lumen_, S_pz,lumen_, correspond to the grayscale values of phase images in the x, y and z directions, while S_mag,lumen_ corresponds to the grayscale value of the final set of magnitude images. To correct for any possible phase offset in the phase image, the average signal intensity of the stationary sternocleidomastoid muscle adjacent to the measured carotid artery was subtracted from the signal intensity of the lumen.

In these PC-MRA images, the Q_CCA_ was measured in MRI slices perpendicular to the luminal centerline. This centerline was established on the magnitude PC-MRA images using 3D Slicer by placing markers at the center of the CCA across nine consecutive slices, beginning 25 mm proximal to the carotid bifurcation. The resulting centerline was then used in a curved planar reformatting, which straightened the image volume along the CCA. Three luminal cross-sections were segmented in this straightened volume on three consecutive slices, approximately 22.5 mm proximal to the bifurcation. Velocities normal to each cross-section were subsequently computed using the following formula:

$$V_{n}=V\cdot U_{n}$$

Here, V_n_ refers to the dot product of the local velocity field (V = [v_x_ v_y_ v_x_]) and U_n_, the normalized unit vector of the three segmented cross-sections. For each cross section, flow was calculated by multiplying the cross-sectional area by the average velocity component normal to the plane:

$$Q=A*V_{n}$$

A mean Q_CCA_ was then obtained by averaging the flow values across the three cross-sections.
