# Supplementary material for: MRI-based shear stress profiles in subclinical carotid atherosclerosis – A feasibility study
Source: PLOS Digit Health. 2026 Aug 4;5(8):e0001618. doi: 10.1371/journal.pdig.0001618 (PMC13436755; doi:10.1371/journal.pdig.0001618)
Supplement: Supplementary file 4 [file pdig.0001618.s004.docx]

**S1 Table. Comparison of measurements between 100 left and 100 right carotid arteries**

|  | Mean (SD) / Median (IQR) | |  |
| --- | --- | --- | --- |
| Characteristic | Left carotid | Right carotid | P-value^a^ |
| Common carotid |  |  |  |
| Diameter, mm | 6.16 (0.32) | 6.36 (0.25) | 0.003 |
| Flow, ml/s | 3.62 (1.19) | 3.47 (1.13) | 0.148 |
| Minimum WSS, Pa | 0.09 (0.05-0.14) | 0.09 (0.06-0.15) | 0.055 |
| 5th percentile WSS, Pa | 0.19 (0.12-0.25) | 0.19 (0.11-0.26) | 0.252 |
| Mean WSS, Pa | 0.53 (0.39-0.70) | 0.45 (0.33-0.60) | 0.001 |
| Maximum WSS, Pa | 1.13 (0.78-1.60) | 0.85 (0.65-1.28) | <0.001 |
| 95th percentile WSS, Pa | 0.90 (0.62-1.27) | 0.68 (0.51-0.93) | <0.001 |
| Internal carotid |  |  |  |
| Diameter, mm | 4.98 (0.71) | 4.94 (0.72) | <0.001 |
| Flow, ml/s | 2.30 (0.76) | 2.20 (0.71) | 0.144 |
| Minimum WSS, Pa | 0.08 (0.05-0.13) | 0.07 (0.03-0.13) | 0.575 |
| 5th percentile WSS, Pa | 0.17 (0.12-0.27) | 0.15 (0.10-0.26) | 0.082 |
| Mean WSS, Pa | 1.08 (0.77-1.52) | 0.96 (0.72-1.36) | 0.061 |
| Maximum WSS, Pa | 5.51 (3.18-6.98) | 3.76 (2.46-5.60) | 0.005 |
| 95th percentile WSS, Pa | 2.59 (1.85-3.77) | 2.26 (1.60-3.10) | 0.016 |
| At the level of MLA |  |  |  |
| Cross-sectional area, mm^2^ | 13.4 (10.8-16.3) | 12.8 (11.0-16.1) | 0.428 |
| WSS preceding, Pa^b^ | 1.41 (0.91-2.41) | 1.41 (0.99-2.02) | 0.358 |
| Max WSS gradient, Pa/mm | 3.85 (2.30-6.37) | 3.04 (1.61-4.99) | 0.071 |

Abbreviations: WSS = wall shear stress, MLA = minimum lumen area.

^a^P-values from paired two-sided t-tests for normally distributed continuous variables, or paired two-sided Wilcoxon signed-rank tests for non-normally distributed continuous variables.

^b^Average WSS of the 3 mm zone proximal to the MLA.
