# Supplementary material for: MRI-based shear stress profiles in subclinical carotid atherosclerosis – A feasibility study
Source: PLOS Digit Health. 2026 Aug 4;5(8):e0001618. doi: 10.1371/journal.pdig.0001618 (PMC13436755; doi:10.1371/journal.pdig.0001618)
Supplement: Supplementary file 5 [file pdig.0001618.s005.docx]

**S2 Table. Pairwise comparison of WSS metrics between 100 left and 100 right carotid arteries, adjusted for carotid diameter and flow**

| Model 1 | | | |
| --- | --- | --- | --- |
| Common carotid | Ratio | Internal carotid | Ratio |
| Minimum WSS | 0.83 ** | Minimum WSS | 1.10 |
| 5th percentile WSS | 0.90 * | 5th percentile WSS | 1.20 ** |
| Mean WSS | 1.06 * | Mean WSS | 1.09 ** |
| Maximum WSS | 1.16 ** | Maximum WSS | 1.22 *** |
| 95th percentile WSS | 1.11 ** | 95th percentile WSS | 1.15 ** |
| Model 2 | | | |
| Common carotid | Ratio | Internal carotid | Ratio |
| Minimum WSS | 0.81 *** | Minimum WSS | 1.07 |
| 5th percentile WSS | 0.87 *** | 5th percentile WSS | 1.16 ** |
| Mean WSS | 0.96 | Mean WSS | 1.04 * |
| Maximum WSS | 1.07 | Maximum WSS | 1.15 *** |
| 95th percentile WSS | 1.04 | 95th percentile WSS | 1.10 ** |

Abbreviations: WSS = wall shear stress

Values represent model-adjusted ratios of WSS metrics between the left and right carotid arteries (ratio = left / right). Model 1 adjusted the metrics for mean diameter of their respective carotid segment. Model 2 additionally adjusted for the flow of the respective segment.

Significance is indicated as follows: * p < 0.05, ** p < 0.01, *** p < 0.001
